# Tooth loss-related dietary patterns and cognitive impairment in an elderly Japanese population: The Nakajima study

**DOI:** 10.1371/journal.pone.0194504

**Published:** 2018-03-15

**Authors:** Mai Ishimiya, Hiroyuki Nakamura, Yutaka Kobayashi, Moeko Noguchi-Shinohara, Chiemi Abe, Chiaki Dohmoto, Yoshihisa Ikeda, Kahori Tokuno, Kazuhiro Ooi, Masami Yokokawa, Kazuo Iwasa, Kiyonobu Komai, Shuichi Kawashiri, Masahito Yamada

**Affiliations:** 1 Department of Oral and Maxillofacial Surgery, Kanazawa University Graduate School of Medical Sciences, Kanazawa, Japan; 2 Department of Neurology and Neurobiology of Aging, Kanazawa University Graduate School of Medical Sciences, Kanazawa, Japan; 3 Department of Physical Therapy, Division of Health Science, Kanazawa University Graduate School of Medical Sciences, Kanazawa, Japan; 4 Department of Neurology, Ioh Hospital, National Hospital Organization, Kanazawa, Japan; Hokkaido Daigaku, JAPAN

## Abstract

Although several studies have demonstrated a potential correlation of dietary patterns with cognitive function, the relationship between tooth loss and dietary patterns and cognitive function have not been identified. In this cross-sectional study, we used a reduced rank regression (RRR) analysis, a technique used previously to observe dietary patterns based on the intakes of nutrients or levels of biomarkers associated with the condition of interest, to identify tooth loss-related dietary patterns and investigate the associations of such patterns with cognitive impairment in 334 community-dwelling Japanese subjects aged ≥ 60 years. According to Pearson correlation coefficients, the intakes of six nutrients (ash content, sodium, zinc, vitamin B1, α- and β-carotene) correlated significantly with the number of remaining teeth. Using RRR analysis, we extracted four dietary patterns in our subject population that explained 86.67% of the total variation in the intakes of these six nutrients. Particularly, dietary pattern 1 (DP1) accounted for 52.2% of the total variation. Food groups with factor loadings of ≥ 0.2 included pickled green leafy vegetables, lettuce/cabbage, green leaves vegetables, cabbage, carrots/squash; by contrast, rice had a factor loading of <−0.2. In a multivariate regression analysis, the adjusted odds ratios regarding the prevalence of cognitive impairment for the lowest, middle and highest tertiles of the DP1 score were 1.00 (reference), 1.224 (95% confidence interval [CI]: 0.611–2.453) and 0.427 (95% CI: 0.191–0.954), respectively. To our knowledge, this is the first report to show that tooth loss-related dietary patterns are associated with a high prevalence of cognitive impairment. These results may motivate changes in dental treatment and the dietary behaviours and thereby lower the risk of cognitive impairment.

## Introduction

Currently, the global population is ageing, and this is accompanied by a rapid increase in the number of patients with dementia [1]. Approximately 46.8 million people worldwide were reported to live with dementia in 2015, and this population is expected to increase to 131.5 million by 2050 [2]. Despite significant research interest, however, the pathogenesis of dementia remains somewhat unclear [3], and few treatments or healthcare options are available for affected patients [4, 5]. These deficiencies indicate the need for in-depth studies that aim to identify factors that could reduce the risk of dementia and consequently reduce the associated healthcare burdens. A previous study demonstrated that a better adherence to a dietary pattern characterised by a high intake of vegetables and low intake of rice correlated with a reduced risk of dementia in a general Japanese population [6]. Several observational longitudinal studies have evaluated the potential relationship between cognitive status and oral health (particularly the number of teeth) [7–18]. However, to the best of our knowledge, there are no studies that have demonstrated the relationship between tooth loss and dietary patterns and cognitive status.

Notably, the intakes of certain foods, including fish and vegetables, were reported to potentially protect against all-cause dementia and Alzheimer’s disease in an epidemiological study [19]. However, the results of studies of the effects of nutrient intakes on cognitive outcomes remain inconclusive [19, 20], and such studies are complicated by the fact that foods and nutrients are consumed in combination (i.e. meals), rather than in isolation. Accordingly, an evaluation of dietary patterns might identify the factors that most strongly protect against the development of dementia. Recently, dietary pattern analyses have been introduced in conjunction with more traditional studies. In such an analysis, a subject’s overall diet is evaluated to determine the effects on disease risks and may be particularly useful when the development of a disease can be attributed to multiple nutrients [21]. A more recent publication proposed reduced rank regression (RRR) as a novel a posteriori statistical method that combines information about a specific disease (e.g. biomarkers) with population-level dietary data (e.g. nutrient intake) to identify dietary patterns associated with the disease of interest. [22]. Accordingly, RRR analysis may be appropriate for investigations of dietary patterns associated with health outcomes in a population [22]. Therefore, in this study, which we believe to be the first of its type, we used RRR analysis to identify tooth loss-related dietary patterns and investigate their associations with cognitive impairment in an elderly Japanese population.

## Methods

### Study procedure and participants

This cross-sectional study was conducted as a part of the Nakajima study in Nakajima, Ishikawa Prefecture, Japan, between 2015 and 2016. The Nakajima study is a population-based cohort study that investigated the association between lifestyle and cognitive function of elderly Japanese residents of Nakajima. Additional information about the design of the Nakajima study has been described previously [23, 24]. There are 2,392 elderly individuals aged ≥60 years in Nakajima. In this study, we targeted all inhabitants aged ≥60 years who were legally residing in the Toyokawa area of Nakajima on the prevalence day (1 April 2015), and 522 inhabitants meeting the age requirement were considered potential candidates. The Nakajima study was supported by Nanao city, and the residence information was used to list target candidates and send invitations for a mass examination. The mass examination for the cognitive functions was conducted at public town halls. The examination included a battery of tests for neurological and cognitive function, whereas the survey inquired about the subjects’ lifestyle habits, medical conditions and neuropsychological function. This study was conducted by trained researchers, including five neurologists, two psychologists, seven nurses, five dentists, one physiotherapist and one occupational therapist. In addition, the residents completed self-administered questionnaires soliciting sociodemographic information (e.g. age, sex) and medical histories (e.g. hypertension, hyperlipidaemia and diabetes mellitus), as previously described [23, 24]. The trained researchers reviewed the completed questionnaires to identify inconsistent or unanswered items. To assess the cognitive function of non-participants, we visited them at home. The in-home survey included the same questionnaires used in the mass examination at public town halls. This phase of the Nakajima study was conducted with the assistance of collaborators of the neighbourhood association (local welfare commissioners and chairmen of the neighbourhood association) with the hope that their cooperation would encourage participation of the reluctant residents. The study protocol was approved by the medical ethics review board of Kanazawa University in Japan, and written informed consent was obtained from all participants, who signed a form stating the study aims and methodology, potential risks and benefits to participants, the voluntary nature of participation and right to withdraw without penalty, and a guarantee of confidentiality and personal data security.

### Cognitive status

Diagnosis of dementia was based on the guidelines of the Diagnostic and Statistical Manual of Mental Disorders, third edition, revised (DSM-III-R) [25], whereas diagnosis of MCI was established according to the International Working Group on general criteria for MCI [26], which state that (1) persons should be judged as abnormal using other modalities besides not fulfilling the DSM-III-R dementia criteria, (2) functional activities of the person are mainly preserved or at least impairment is minimal and (3) the person should have evidence of cognitive decline, either by self-assessment and/or the use of an informative report in conjunction with deficits on the objective cognitive tasks. Among participants without dementia, a CDR score of 0.5 was used as the objective cognitive impairment value to denote cognitive and functional impairment consistent with MCI.

### Evaluation of tooth loss

Each patient underwent a clinical oral examination performed by a dentist in accordance with the methodology of the Third National Health and Nutrition Examination Survey [27]. The remaining teeth were classified as healthy, carious, or treated (including crowned, inlay and abutment teeth for prostheses) inclusive of completely erupted third molars. The number of remaining teeth was also recorded as a measure of tooth loss (excluding unerupted or congenitally missing teeth, root tips and extremely mobile teeth indicated for extraction) and grouped into three categories (≥20 or ≤19 with denture, 1–19 without denture, 0 without denture) to indicate the extent of tooth loss with evaluation of denture use.

### Dietary assessment

Each subject completed a validated brief self-administered diet history questionnaire (BDHQ) for the assessment of their dietary habits during the previous 1 month [28]. The BDHQ comprises of five sections soliciting the following information: (I) intake frequencies of 46 foods and non-alcoholic beverages; (II) daily intakes of rice and miso soup; (III) frequencies of overall alcoholic beverage consumption and of the intake of five alcoholic beverages per typical drinking occasion; (IV) frequently used cooking methods; and (V) general dietary behaviours. Subsequently, an ad hoc computer algorithm was used to calculate the dietary intakes of 58 food and beverage items, intake of energy and intakes of 96 nutrients according to the responses to the BDHQ [29], as well as the Standard Tables of Food Composition in Japan [30, 31]. A BDHQ validation study based on 16-day weighted dietary records yielded standard correlation coefficients exceeding 0.40 for the intakes of various foods, beverages [29] and nutrients evaluated in the present study [28].

### Statistical analyses

The associations of dietary patterns with tooth loss were assessed using a RRR analysis [22], and we selected the six nutrients found to correlate significantly with the number of remaining teeth in the categories (≥20 or ≤19 with denture, 1–19 without denture, 0 without denture) as response variables. Subsequently, we derived dietary patterns based on intakes of these six nutrients in 58 food and beverage items. We used the SAS procedure PLS (partial least squares) with the options for RRR analysis. In general, principal component analysis finds combinations of the predictors with large variance. However, this technique makes no use of response values. On the other hand, multiple linear regression finds a combination of the predictors that best fit a response. The RRR technique is something of a cross between principal component analysis and multiple linear regression. The RRR analysis extracts predictors that explain as much response variation as possible with large variance. For further details concerning other possible options and their SAS syntax, refer to the chapter of The PLS Procedure in SAS/STAT 9.3 User’s Guide [32]. The density method was used to energy-adjust all nutrients and food/beverage items before performing the RRR analysis. We additionally calculated the explained percentages of variations and Pearson correlation coefficients between the intakes of nutrients (response variables) and food/beverage items and the extracted dietary patterns; this information was subsequently used to compute dietary pattern scores, which were categorised into tertiles.

A one-way analysis of variance (ANOVA) and chi-square test were applied to evaluate continuous and categorical variables, respectively. Univariate and multivariate logistic regression models were used to determine the independent effects of the number of remaining teeth or dietary pattern score on the risk of cognitive dysfunction (dementia or MCI); here, the lowest category in each measure served as the reference group. In the multivariable-adjusted model, 6 covariates known to be potential risk for dementia were selected: age; sex; history of hypertension, diabetes mellitus, hyperlipidemia; low education, as described in previous Nakajima Study [33, 34]. For these analyses, Model 1 was sex- and age-adjusted, while Model 2 comprised Model 1 with further adjustments for a history of hypertension, diabetes mellitus, hyperlipidaemia and formal education.

For all analyses, statistical significance was defined as a two-sided P-value of < 0.05. SPSS version 23.0J (SPSS Inc., Chicago, IL, USA) or SAS (on-demand academics version; SAS Institute, Cary, NC, USA) was used to perform the statistical analyses.

## Results

**Fig 1:**
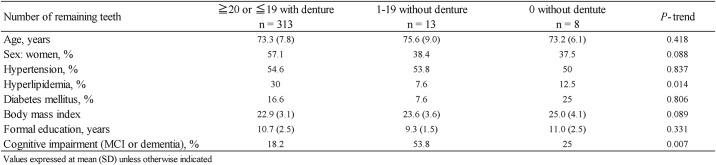
Characteristics of study population based on the number of remaining teeth.

Of the 522 potential candidates, 20 were excluded (4 died and 16 relocated). Thus, the remaining 502 residents were considered as baseline candidates. In total, 214 candidates participated in the mass examination for cognitive function at the public town halls, whereas 215 candidates participated in the in-home survey. In addition, 38 institutionalised individuals were enrolled. In total, 467 residents participated in the present study, and 35 residents refused to participate in our study. We excluded 133 subjects: those with psychiatric illnesses (n = 1), consciousness disturbances (n = 2) and cerebral palsy (n = 2); those who failed to complete the cognitive tests (n = 8) and self-administered questionnaires (n = 53) or those who refused an oral examination (n = 67). Finally, data from 334 subjects were analysed for the prevalence of dementia. The characteristics of the study population according to the number of remaining teeth are shown in Fig 1. Notably, we observed significant differences in the frequencies of hyperlipidaemia and cognitive impairment across the categories for the number of remaining teeth.

**Fig 2:**
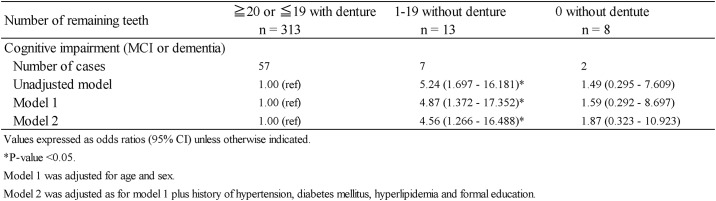
Association between the number of remaining teeth and cognitive impairment.

Fig 2 demonstrates the inverse association of the number of remaining teeth with the prevalence of cognitive impairment (dementia or MCI) models adjusted for age-, sex, hypertension, diabetes mellitus, hyperlipidaemia and formal education. The multivariate-adjusted odds ratios regarding the prevalence of cognitive impairment were 1.00 (reference) for ≥ 20 teeth or ≤19 with dentures, 4.56 (95% confidence interval [CI]: 1.266–16.488; P = 0.02) for 1–19 teeth without dentures and 1.87 (95% CI: 0.323–10.923; P = 0.482) for 0 teeth without dentures (Fig 2).

**Fig 3:**
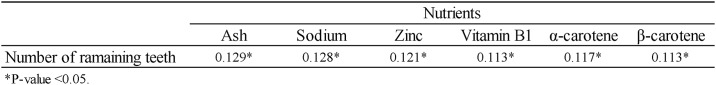
Pearson’s correlation coefficients for the number of remaining teeth nutrient intake.

Fig 3 lists the Pearson correlation coefficients of nutrient intakes with the number of remaining teeth. In this study, we observed significant correlations between the number of remaining teeth and the intakes of six nutrients (ash content, sodium, zinc, vitamin B1, α-carotene and β-carotene) selected from among a panel of 96 nutrients; these six nutrients were designated as response variables. We analysed the differences between two categories for the number of remaining teeth (≥20 and ≤19 with denture). However, there were no significant correlations between the number of remaining teeth in these categories (≥20 and ≤19 with denture) and the intake of 96 nutrients. In addition, there were no significant associations between the number of remaining teeth in these categories (≥20 and ≤19 with denture) and the prevalence of cognitive impairment models adjusted for age, sex, hypertension, diabetes mellitus, hyperlipidaemia and formal education. Subsequently, in this study, the two categories for the number of remaining teeth (≥20 and ≤19 with denture) were combined into one category.

**Fig 4:**
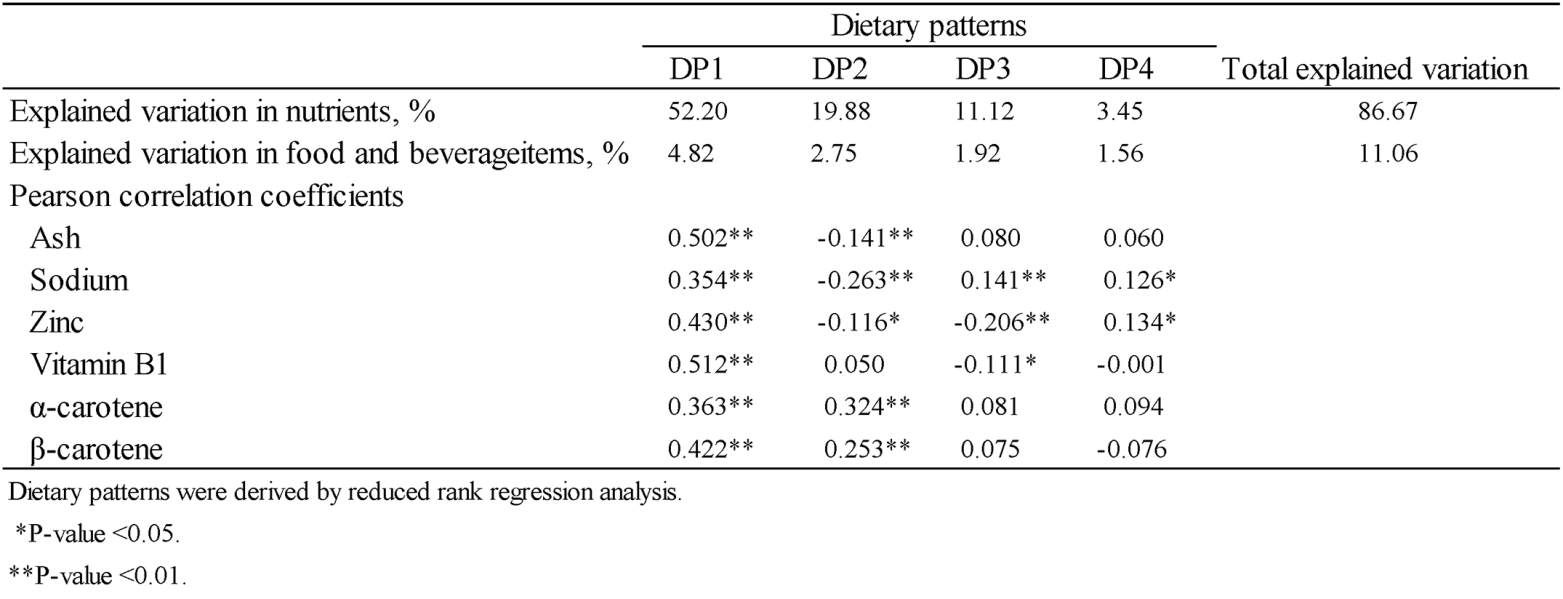
Explained percentage of variation in nutrients (response variables) and food and beverage items by extracted dietary patterns.

Subsequently, we used the RRR analysis to extract four dietary patterns in our participant sample that taken together, explained 86.67% of the total variation in the intakes of the response variables (Fig 4). As dietary pattern 1 (DP1) accounted for 52.2% of the total variation whereas DP2–4 explained very few of the observed variations (Fig 4), we selected the DP1 scores as the target dietary pattern in further analyses. In a Pearson’s correlation analysis, we identified strong correlations of the DP1 scores with the intakes of all six nutrients identified as response variables (Pearson’s correlation coefficients <0.01 for all; Fig 4).

**Fig 5:**
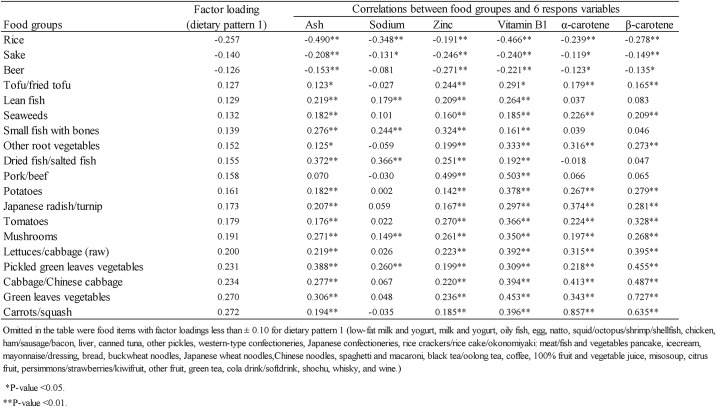
Factor loadings of food groups associated with dietary pattern 1 and correlation coefficients between food groups and nutrients (response variable).

Fig 5 presents factor loadings indicating the magnitudes and directions of the contributions of 19 food groups to the DP1 scores, as well as the correlation coefficients of these food groups with the six previously identified response variables. Notably, a positive factor loading value indicates an association between an increased intake of that food with a higher DP1 score. We identified the following foods with factor loadings ≥ 0.2: pickled green leafy vegetables, lettuce/cabbage (raw), green leafy vegetables, cabbage/Chinese cabbage and carrots/squash. By contrast, rice had a factor loading of < -0.2.

**Fig 6:**
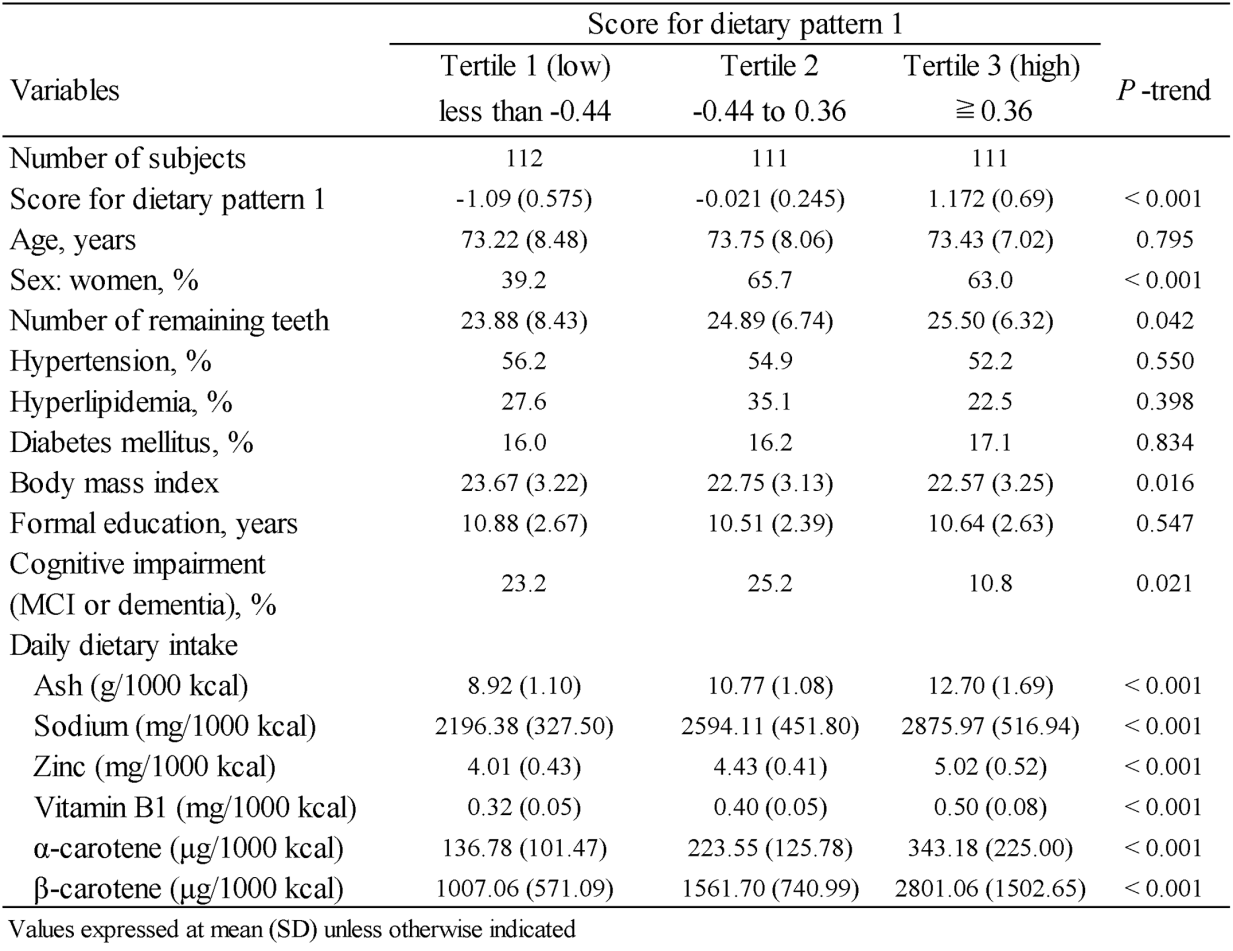
Characteristics of the study population by tertiles of dietary pattern 1 scores.

Fig 6 presents the clinical characteristics of the study subjects by DP1 score tertiles. Subjects with lower DP1 scores were more likely to be men, to exhibit cognitive impairment (dementia or MCI) and to have a higher mean body mass index. A higher DP1 score was associated with number of remaining teeth and higher mean intakes of ash content, sodium, zinc, vitamin B1, α-carotene and β-carotene.

**Fig 7:**
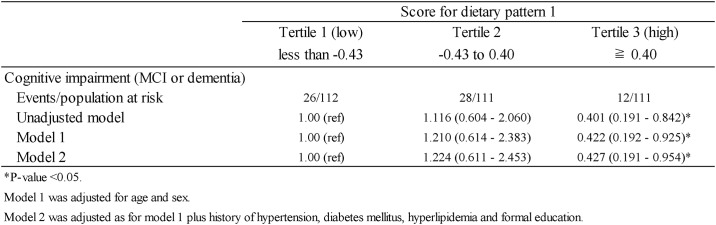
Odds ratios and 95% confidence intervals for cognitive impairment based on tertiles of dietary pattern 1 scores.

The odds ratios of cognitive impairment (dementia or MCI) according to DP1 tertile category are shown in Fig 7. In Model 1, DP1 tertile 3 (≥0.40) was significantly associated with a decreased prevalence of cognitive impairment. Although this association was somewhat attenuated by further adjustments for other covariates (Model 2), it remained significant. The multivariate-adjusted odds ratios for the lowest, middle and highest DP1 tertiles were 1.00 (reference), 1.224 (95% CI: 0.611–2.453; P = 0.568) and 0.427 (95% CI: 0.191–0.954; P = 0.038), respectively.

## Discussion

The present study aimed to evaluate the relationship between tooth loss and cognitive impairment, in light of several similar prospective studies of the risk of all-cause dementia associated with tooth loss [9–12, 15]. The findings of a prospective cohort study of elderly Japanese adults, in which subjects with few remaining teeth and no dentures had a significantly higher incidence of all-cause dementia relative to those retaining at least 20 teeth [9], agreed well with our current findings of a significantly higher prevalence of cognitive impairment (dementia or MCI) in subjects with fewer teeth without denture. However, we and others failed to identify a significant difference between subjects with no teeth without dentures and those with 20 teeth or less than 19 with a denture [10, 12, 15]. These discrepancies suggest the existence of currently unknown mechanisms underlying the association between tooth loss and cognitive impairment.

To better clarify this relationship, we conducted the first RRR analysis [22] of dietary patterns associated with tooth loss in the current study. Previous studies have generally based such analyses on a hypothesis-oriented approach involving a known ideal dietary pattern (e.g. Mediterranean diet) or a principal component analysis in which dietary patterns specific to the target population are identified. By contrast, the RRR analysis does not require the identification of an already-known dietary pattern and can incorporate existing data concerning the relationship between the diet and disease of interest, as it uses the linear functions of food groups within a dietary pattern to best explain variations in of nutrients identified as risk-related or preventive factors for the disease of interest. We determined that the RRR analysis would likely be most appropriate for estimating tooth loss-related dietary patterns, as the scores generated using this method would likely correlate with tooth loss.

From our survey, we identified the following six nutrients (among a panel of 96) as significantly related with the number of remaining teeth: ash content, sodium, zinc, vitamin B1, α-carotene and β-carotene and derived dietary patterns related to the intakes of these nutrients based on 58 food groups. Accordingly, we identified that a dietary pattern characterised by high intakes of pickled green leafy vegetables, lettuce/cabbage (raw), green leafy vegetables, cabbage/Chinese cabbage and carrots/squash (i.e. DP1). Notably, in our study, the highest DP1 score tertile was significantly associated with a decreased prevalence of cognitive impairment. We further identified a negative correlation of the intakes of rice, sake and beer with DP1, thus suggesting an association of this pattern with an increased prevalence of cognitive impairment. A previous observational longitudinal study demonstrated that a better adherence to a dietary pattern characterised by a low intake of vegetables and high intake of rice, similar to the tooth loss-related dietary pattern in this study, correlated with an increased risk of dementia in a general Japanese population [6]. We note that people with few or no teeth and no dentures will find it difficult to consume vegetables, whereas rice, sake and beer would be more easily ingested. Therefore, our findings suggest that a low DP1 score was associated with a high prevalence of cognitive impairment, and tooth loss may be directly linked with the low DP1 score. The factors increasing the risk of tooth loss are poor oral hygiene and subsequent dental caries and periodontal disease [35]. Patients with cognitive impairment are at an increased risk of poor oral hygiene [36] and may develop a tooth loss-related dietary pattern. However, sugar intake has long been established as the major contributing factor in dental caries and periodontal disease [35]. Because sugar was not identified as nutrient related with the number of remaining teeth, the poor dietary pattern in the present study may not be a factor toward prevalence of tooth loss. These suggestions underscore the importance of appropriate and accessible dental care and treatment. Specifically, dental maintenance early in life or even after diagnosis of mild cognitive impairment might be an important step toward reducing the long-term risk of dementia. Further prospective longitudinal studies are necessary to reveal that tooth loss-related dietary patterns are associated with a risk of dementia.

We note that the current study had some limitations, including cross-sectional study in the relatively small sample size. Additionally, we did not use diagnostic tools (e.g. neuroimaging, neuropathology) to evaluate the causes of dementia and MCI in the study participants and note that additional studies involving these tools are needed to determine the true underlying causes of cognitive impairment in this population. Another limitation of the study is that the diet of our study population cannot be generalised to other populations, wherein common dietary patterns would consist of different nutrients and foods. Furthermore, we did not observe a high rate of valid responses at baseline in our subject population. Despite these limitations, however, the current study benefitted from the adjustment of potential confounding factors in the analytical models.

In conclusion, we showed that a tooth loss-related dietary pattern was associated cognitive impairment in a general Japanese population. Our findings suggest that better adherence to a dietary pattern characterised by a high intake of pickled green leafy vegetables, lettuce/cabbage (raw), green leafy vegetables, cabbage/Chinese cabbage and carrots/squash and a low intake of rice correlates positively with the number of remaining teeth and negatively with the cognitive impairment. Our findings may motivate changes in the dental treatment and the dietary behaviour within the general Japanese population, thereby, lowering the risk of dementia.

## Abbreviations

CI: confidence interval
DP: dietary pattern
OR: odds ratio
RRR: reduced rank regression
